# TLR2 and interleukin-10 are involved in *Bacteroides fragilis*-mediated prevention of DSS-induced colitis in gnotobiotic mice

**DOI:** 10.1371/journal.pone.0180025

**Published:** 2017-07-06

**Authors:** Yi-Chih Chang, Yung-Hao Ching, Chien-Chao Chiu, Ju-Yun Liu, Shao-Wen Hung, Wen-Ching Huang, Yen-Te Huang, Hsiao-Li Chuang

**Affiliations:** 1 Department of Medical Laboratory Science and Biotechnology, China Medical University, Taichung, Taiwan; 2 Department of Molecular Biology and Human Genetics, Tzu Chi University, Hualien, Taiwan; 3 Division of Animal Resources, Animal Technology Laboratories, Agricultural Technology Research Institute, Hsinchu, Taiwan; 4 National Laboratory Animal Center, National Applied Research Laboratories, Taipei, Taiwan; 5 Department of Exercise and Health Science, National Taipei University of Nursing and Health Sciences, Taipei, Taiwan; "INSERM", FRANCE

## Abstract

**Background and aims:**

*Bacteroides fragilis* (*BF*) are Gram-negative anaerobe symbionts present in the colon. Recent studies have reported the beneficial role of *BF* in maintaining intestinal homeostasis, stimulating host immunologic development, and preventing infectious colitis caused by pathogenic bacteria. Our previous studies showed that monocolonization of germ-free mice with *BF* significantly reduced colon inflammations and damage.

**Methods:**

In order to investigate the Toll-like receptor-2 (TLR2), TLR4, and interleukin 10 (IL-10) molecular signaling pathways involved in *BF*-mediated prevention of dextran sulfate sodium (DSS)-induced colitis. The wild-type (WT), TLR4, TLR2, and IL-10 knockout (^-/-^) germ-free mice grown were with or without *BF* colonization for 28 days, and then administered 1% DSS in drinking water for 7 day to induce acute ulcerative colitis.

**Results:**

We compared phenotypes such as weight loss, disease activity, intestinal histological scores, and immunohistochemistry for inflammatory cells. Unlike WT and TLR4^-/-^ mice, the severity of DSS-colitis did not improve in TLR2^-/-^ animals after *BF* colonization. The *BF* enhanced anti-inflammatory cytokines IL-10 expression and inhibited pro-inflammatory-related tumor necrosis factor (TNF-α) and IL-6 mRNA expression in both WT and TLR4^-/-^ mice. In contrast, the failed to up-regulated IL-10 and down-regulated the TNF-α and IL-6 in *BF* colonization TLR2^-/-^ mice. In addition, we further perform IL-10^-/-^ mice to clarify whether the BF through TLR2 /IL-10 pathway to alleviate DSS-colitis. There were no significant differences in colitis severity and pro-inflammatory related genes expression in the IL-10^-/-^ mice with or without *BF* colonization.

**Conclusions:**

These results indicate the disease-preventing effects of *BF* in acute DSS-induced colitis may occur through the TLR2/IL-10 signal pathway.

## Introduction

Inflammatory bowel diseases (IBD) are characterized by chronic and repeated inflammatory disorders of the gut including Crohn’s disease (CD) and ulcerative colitis (UC); however, their etiologies remain unknown [1, 2]. The pathogenesis of UC is associated with a complex interplay of genetic, immune system, microbial, and environmental factors [3]. It is generally accepted that IBDs are associated with dysregulation of the mucosal immune system in the native gut microbiota in genetically susceptible individuals. Many studies demonstrated that the gut microbial composition is the strongest environmental factor affecting IBD prognosis [4, 5]. Current treatments for IBD are restricted to the use of anti-inflammatory drugs, immunosuppressants, and antibiotics [6]. The DSS-induced experimental murine colitis model is well-established and shows consistent results in studies of profound inflammatory changes that occur in the gut during disease progression [7–9]. Previous studies suggested that CD is a T helper 1-mediated immune disease characterized by increased cytokine levels of IFN-γ, TNF-α, and IL-12, whereas UC is a T helper 2-mediated immune disease characterized by increased cytokine levels of IL-6 and/or reduced IL-10 [10].

*Bacteroides* spp. are an important component of the mammalian gut commensal bacteria and maintain a complex and generally beneficial relationship with the host by performing immunoregulatory, energy metabolic, and physiologic homeostasis functions [11, 12]. Recent studies reported that *Bacteroides fragilis* (*BF*) has profound beneficial effects on the host immune system [11]. Moreover, *BF* protected against experimental colitis induced by trinitrobenzene sulfonic acid, *Helicobacter* spp., and DSS by suppressing the activity of inflammatory-related molecules and inducing the production of anti-inflammatory cytokines [8, 13, 14].

Toll-like receptors (TLRs) are members of the pattern recognition receptor family and are characterized by an extracellular or luminal ligand-binding domain containing leucine-rich repeat motifs and a cytoplasmic signaling Toll/interleukin-1 (IL-1) receptor homology domain. Bacterial lipopeptides are recognized by TLR2 and lipopolysaccharides are recognized by TLR4 [15]. Therefore, TLR2, TLR4, and gut microbial flora play important roles in the process of UC [16]. Dong et al. reported higher expression of TLR2 and TLR4 in colonic epithelial cells during DSS-induced colitis [17]. Similarly, upregulated expression of TLR2 and TLR4 in biopsy samples of patients with IBD was observed [18]. Treatment with TLR2 and TLR4 monoclonal antibodies were shown to significantly decrease gene expression [17]. In addition, treatment with TLR2 ligands ameliorated crypt damage and accelerated healing during DSS administration [19, 20]. Recent studies have demonstrated the critical role of TLR/MyD88 in promoting the differentiation of Th1 cells and development of spontaneous colitis in IL-10^−/−^ mice [21, 22].

GF animal models have been valuable tools in investigating the effects of restricted intestinal bacteria [23]. Probiotics (*Lactobacillus* and *Bifidobacterium*) were shown to reduce tissue damage and the levels of inflammatory cytokines in a model of IBD [24, 25]. Our previous study showed that in GF mice, colonization with *BF* protected against DSS-induced colitis [8]. This was because of decreases in the expression of inflammatory-related molecules and increases in anti-inflammatory cytokine molecules. However, the role of *BF* in maintaining intestinal innate immune system homeostasis and regulating inflammatory responses in DSS-colitis remain unclear. In this study, we utilized WT, TLR2, and TLR4 GF mice colonized with *BF* for 28 days. We subsequently administered 1% DSS and assessed colitis severity. Our results showed that *BF* ameliorated DSS-induced colitis through the TLR2 signal pathway, but not the TLR4 pathway. Moreover, DSS-colitis was not ameliorated in IL-10 knockout mice with *BF* colonization. We suggest that *BF* prevents DSS-colitis via the TLR2/IL-10 signal pathway.

## Materials and methods

### Animals

GF male C57BL/6JNarl (wild-type, WT), B6.129-*Tlr2*^*tm1Kir*^/J (TLR2), B6.B10ScN-*Tlr4*^*lps-del*^/JthJ (TLR4) and B6.129P2-*Il10*^*tm1Cgn*^/J (IL-10) mice (7–8 weeks old) were obtained from National Laboratory Animal Center (Taipei, Taiwan) and purchased from the Jackson Laboratory (Bar Harbor, ME, USA). Mice were maintained in a vinyl isolator in a room kept at a constant temperature (21 ± 1°C) and humidity (55–65%) with a 12-h/12-h, light/dark schedule. Mice were fed a commercial diet (5010 LabDiet, Purina Mills, St. Louis, MO, USA) and sterile water *ad libitum*. To confirm GF status, microbiological assays were performed every month by culturing the feces, bedding, and drinking water in thioglycollate medium (DIFCO, Detroit, MI, USA).

### Ethics statement

All procedures were performed in an animal facility accredited by the Association for Assessment and Accreditation for Laboratory Animal Care, with the approval of the Institutional Animal Care and Use Committee at National Laboratory Animal Center who approved the mouse experiments with the approval number IACUC2011M04. The methods applied in this study were carried out in accordance with approved guidelines.

### Bacterial culture and monocolonization in mice

*BF* strain NCTC 9343 was obtained from the Food Industry Research and Development Institute (Hsinchu, Taiwan). Bacterial cultures were grown overnight in thioglycollate medium at 37°C in an anaerobic incubator, collected by centrifugation (3 min at 2000 ×*g*), and washed 3 times with phosphate-buffered saline. Pellets were re-suspended in 20 mL sterile saline. *BF* was introduced into mice by oral gavage with 5 × 10^7^ colony-formation units in 0.5 mL saline. Control mice were treated with the same volume of sterile saline.

### Induction of experimental colitis in mice

The acute ulcerative colitis mice model was induced by the addition of 1% DSS (36–50 kDa, MP Biomedicals, OH, USA) in sterile filtered drinking water for 7 days. DSS solution was replaced with fresh DSS every 2 days. Dehydration and weight loss are common symptoms of acute colitis. In this study, the animals were not severely ill and did not die prior to the experimental endpoint. In the first experiment, the mice were randomly divided into the following 12 groups (n = 8 each) for treatment: distilled drinking water (WT/GF/WA, TLR2/GF/WA, and TLR4/GF/WA) or 1% DSS in drinking water (WT/GF/DSS, TLR2/GF/DSS, and TLR4/GF/DSS); *BF* monocolonization for 28 days, and then distilled drinking water (WT/*BF*/WA, TLR2/*BF*/WA, and TLR4/*BF*/WA) or 1% DSS in drinking water (WT/*BF*/DSS, TLR2/*BF*/DSS, and TLR4/*BF*/DSS). In the second experiment, IL-10 knockout mice were randomly divided into 2 groups (n = 8 each). The 1% DSS in drinking water was added without *BF* colonization (10/GF/DSS); *BF* monocolonization for 28 days, and then 1% DSS in drinking water (10/*BF*/DSS). At day 7 after DSS treatment, the mice were sacrificed by carbon dioxide inhalation and the body weight, spleen weight, and colon length were measured.

### Complete blood cell count analysis

Blood was collected by intracardiac puncture and treated with ethylenediaminetetraacetate. Total blood cells, differential leukocytes, erythrocytes, hemoglobin level, platelets, lymphocytes, and neutrophils were measured using the Bayer Hematology System (ADVIA 2010, Bayer, Leverkusen, Germany).

### Occult blood assessment in stools

The presence of blood in stools was assessed by the occult blood reagent method (Shih-Yung Medical, Taipei, Taiwan) and scored on a 0–4 point scale: 0, negative; 1, faint blue; 2, moderately blue; 3, dark blue; and 4, fecal blood visible to the eye.

### Histopathology

Mouse colons were fixed in 10% neutral buffered formalin, embedded in paraffin using a standard protocol, cut into 4-μm sections, stained with hematoxylin and eosin, and assessed by light microscopy. Colon sections were scored as grade 0: normal mucosa, grade; 1: slightly infiltration of inflammatory cells within disrupted colonic epithelium; grade 2: mild inflammatory cells infiltration and shortening of the crypt; grade 3: moderate inflammatory cells infiltration, crypt loss, and submucosa edema appearance; grade 4: severe inflammatory cells infiltration and submucosa edema, destruction of epithelial cells (ulceration and erosion). The histopathologic score was determined by a veterinary pathologist blinded to the classification of each mouse.

### Immunohistochemical analysis

Paraffin-embedded colon sections were deparaffinized, rehydrated, and subjected to antigen retrieval, and then incubated with 3% H_2_O_2_ to eliminate endogenous peroxidase activity. Sections were incubated with 10% skin milk to reduce nonspecific reactions and incubated at 4°C overnight with rat anti-mouse monoclonal antibodies against F4/80 (1:50; BioLegend, San Diego, CA, USA) or goat anti-mouse polyclonal antibody specific to Ly-6G^+^ (1:100; Santa Cruz Biotechnology, Santa Cruz, CA, USA). Samples were then incubated with horseradish peroxidase-conjugated anti-rat (HRP Polymer Conjugate, Invitrogen, Carlsbad, CA, USA) or anti-goat antibodies; signals were detected by adding a chromogenic substrate. Sections were rinsed with deionized water, counterstained with hematoxylin, and mounted for histological analysis.

### Real-time PCR analysis of inflammation-related genes

Total RNA was isolated from colon tissue using the RNeasy Minikit (Qiagen, Hilden, Germany). First-strand complementary DNA was synthesized using the Transcriptor First Strand cDNA synthesis kit (Roche Diagnostics, GmbH, Basel, Switzerland). Quantitative real-time PCR were conducted using the TaqMan gene expression assay (Universal Probe Library, Roche Diagnostics GmbH) with a LightCycler 1.5 (Roche Diagnostics GmbH) as follows: 95°C for 10 min, followed by 40 cycles of 95°C for 10 s, 60°C for 25 s, and 40°C for 30 s. β-Actin was used as an internal control and nuclease-free water served as the negative control. The sequences of primers used for analysis are listed in Table 1. The comparative Ct method was used to evaluate relative mRNA levels in the colon tissue.

**Table 1:** Real-time PCR primers used in this study.

Gene (GenBank ID)	Orientation	Sequence (5′-3′)	UPL
TNF-α (NM_013693.2)	Forward	tgcctatgtctcagcctcttc	49
	Reverse	gaggccatttgggaacttct	
IL-10 (NM_010548.1)	Forward	cagagccacatgctcctaga	41
	Reverse	tgtccagctggtcctttgtt	
IL-6 (NM_031168.1)	Forward	gctaccaaactggatataatcagga	6
	Reverse	ccaggtagctatggtactccagaa	
β-actin (NM_007393.3)	Forward	ctaaggccaaccgtgaaaag	64
	Reverse	accagaggcatacagggaca	

### Statistical analysis

Statistical analyses were performed using Graph-Pad Prism 6 software (GraphPad, Inc., La Jolla, CA, USA). The results are presented as the mean ± SD. Differences between groups were analyzed using a non-parametric Mann-Whitney U test or one-way analysis of variance with Bonferroni correction for multiple comparisons. A *P*-value of less than 0.05 was considered significant.

## Results

### Disease symptoms

**Fig 1:**
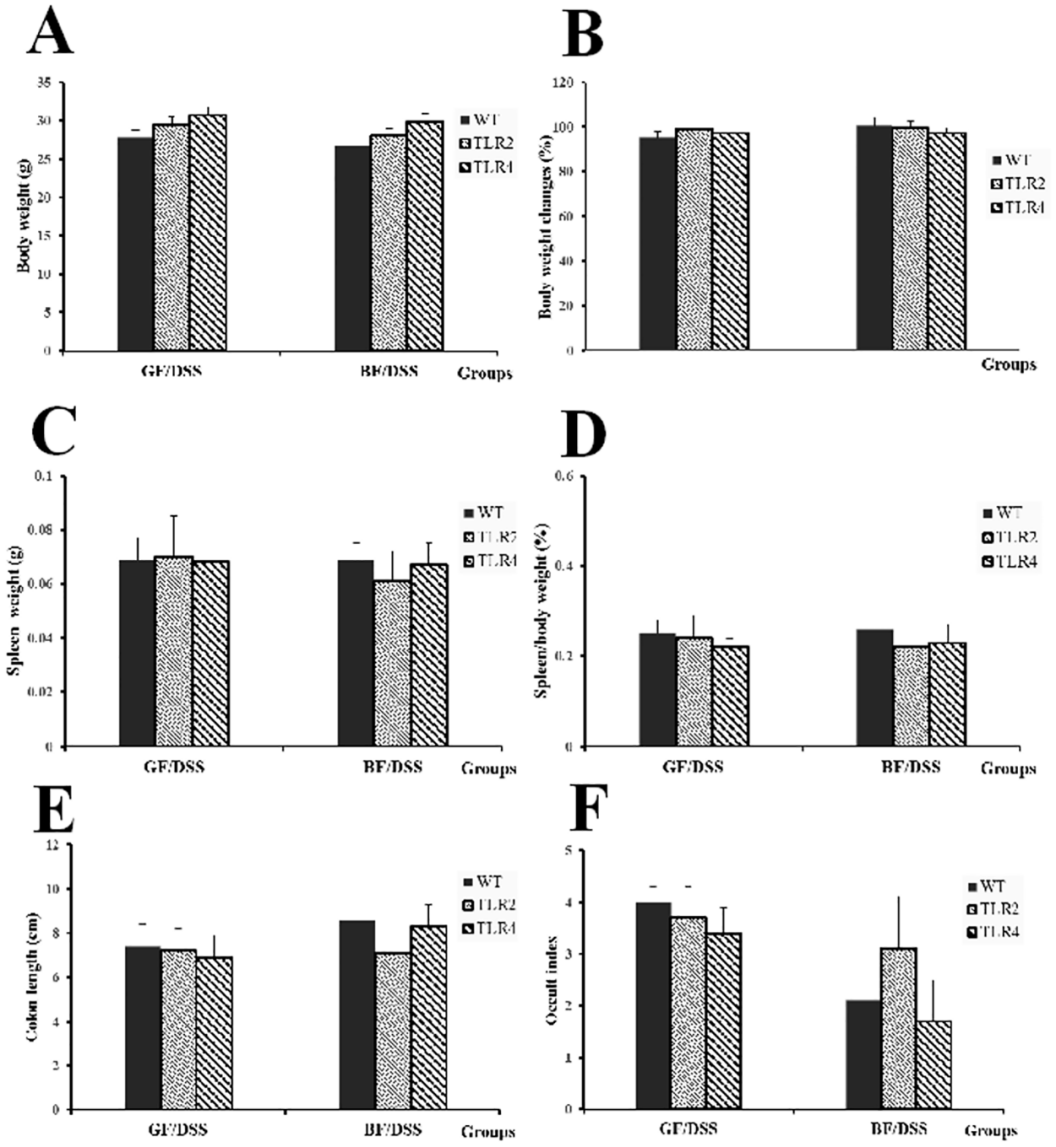
DSS-related clinical and gross alteration in WT, TLR4, and TLR2 GF-mice with or without *BF* colonization. (A) Body weight, (B) body weight changes, (C) spleen weight, (D) spleen/body weight, (E) colon length, (F) occult index. *P < 0.05 compared to WT/GF/DSS.

As shown in S1 Table, mono-colonization of *BF* did not cause any clinical signs or gross alterations in WT, TLR2, and TLR4 mice. Upon administration of DSS, mice in the WT/GF/DSS, TLR4/GF/DSS, and TLR2/GF/DSS groups developed severe colitis and colitis-related signs (based on colon lengths and fecal occult tests). Fecal occult was tested at the end of the experiment. Interestingly, mono-colonization of *BF* bacteria reduced fecal occult blood in the WT/*BF*/DSS group. Similar changes were observed in the TLR4/*BF*/DSS group. However, *BF* colonization had no impact on fecal occult blood severity in the TLR2/*BF*/DSS group over the 7-day experimental period. The severity of colitis was associated with a significantly shorter colon length in WT/GF/DSS compared to that of WT/*BF*/DSS mice (*P* < 0.05). Administration of *BF* improved DSS-induced effects on colon length in the TLR4/*BF*/DSS group, but not in the TLR2/*BF*/DSS group. There were no differences in the body and spleen weights among groups (Fig 1).

### Complete blood count

**Fig 2:**
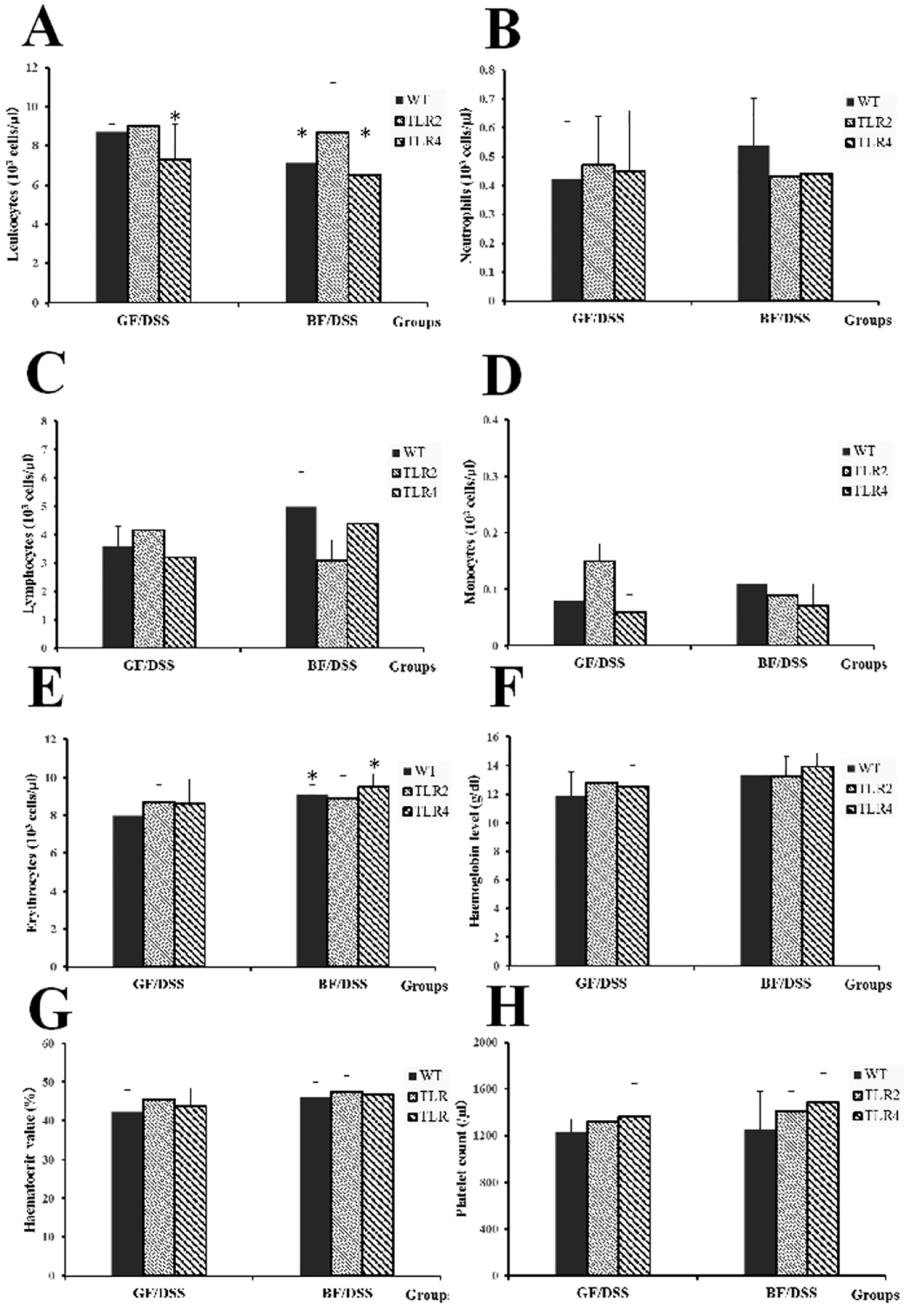
DSS-induced hematological characteristics in WT, TLR4, and TLR2 mice under GF- and *BF*-condition. (A) Leukocytes, (B) neutrophils, (C) lymphocytes, (D) monocytes, (E) erythrocytes, (F) hemoglobin level, (G) hematocrit value, (H) platelet. *P < 0.05 compared to WT/GF/DSS.

There were no differences in the anemic evaluation and leukocyte counts after *BF* colonization without DSS administration (S1 Table). Seven days after administration of DSS to GF mice, three animals became anemic, as indicated by decreased erythrocyte counts and hemoglobin levels. There were no significant differences between groups. However, the erythrocyte counts and hemoglobin levels were higher in the WT/*BF*/DSS and TLR4/*BF*/DSS groups than in the TLR2/*BF*/DSS group. Administration of DSS also induced elevated leukocyte counts in the three GF condition groups. Interestingly, leukocyte counts were significantly lower in the WT/*BF*/DSS and TLR4/*BF*/DSS groups than in the TLR2/*BF*/DSS group (Fig 2).

### Histopathology

**Fig 3:**
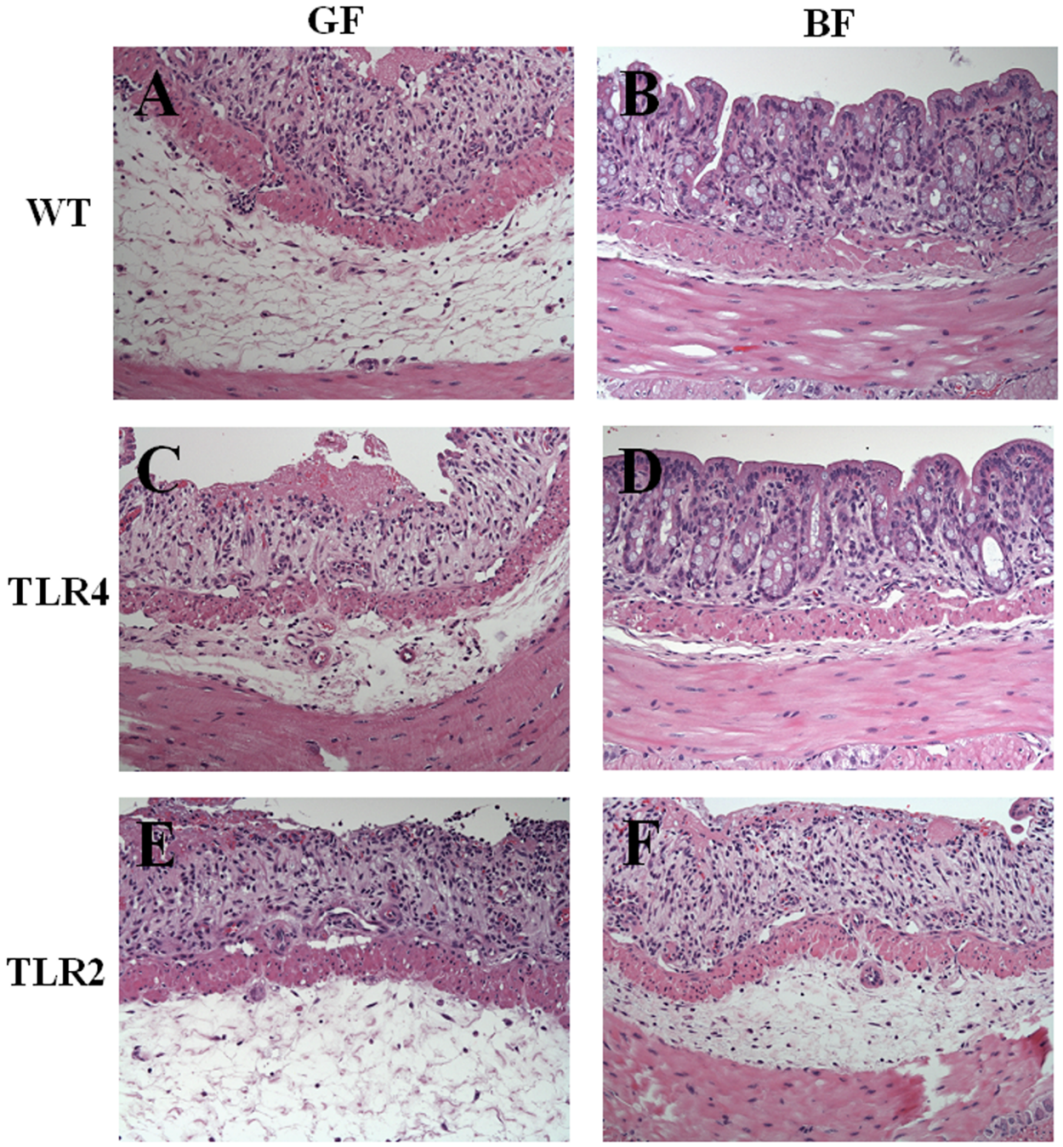
Histopathology revealed the effect of *BF* monocolonization on DSS-induced colon damage in WT, TLR4, and TLR2 mice at day 7. (A) WT/GF/DSS, (B) WT/*BF*/DSS, (C) TLR4/GF/DSS, (D) TLR4/*BF*/DSS, (E) TLR2/GF/DSS, and (F) TLR2/*BF*/DSS. H&E, magnification ×200, bar = 20 *μ*m.

**Fig 4:**
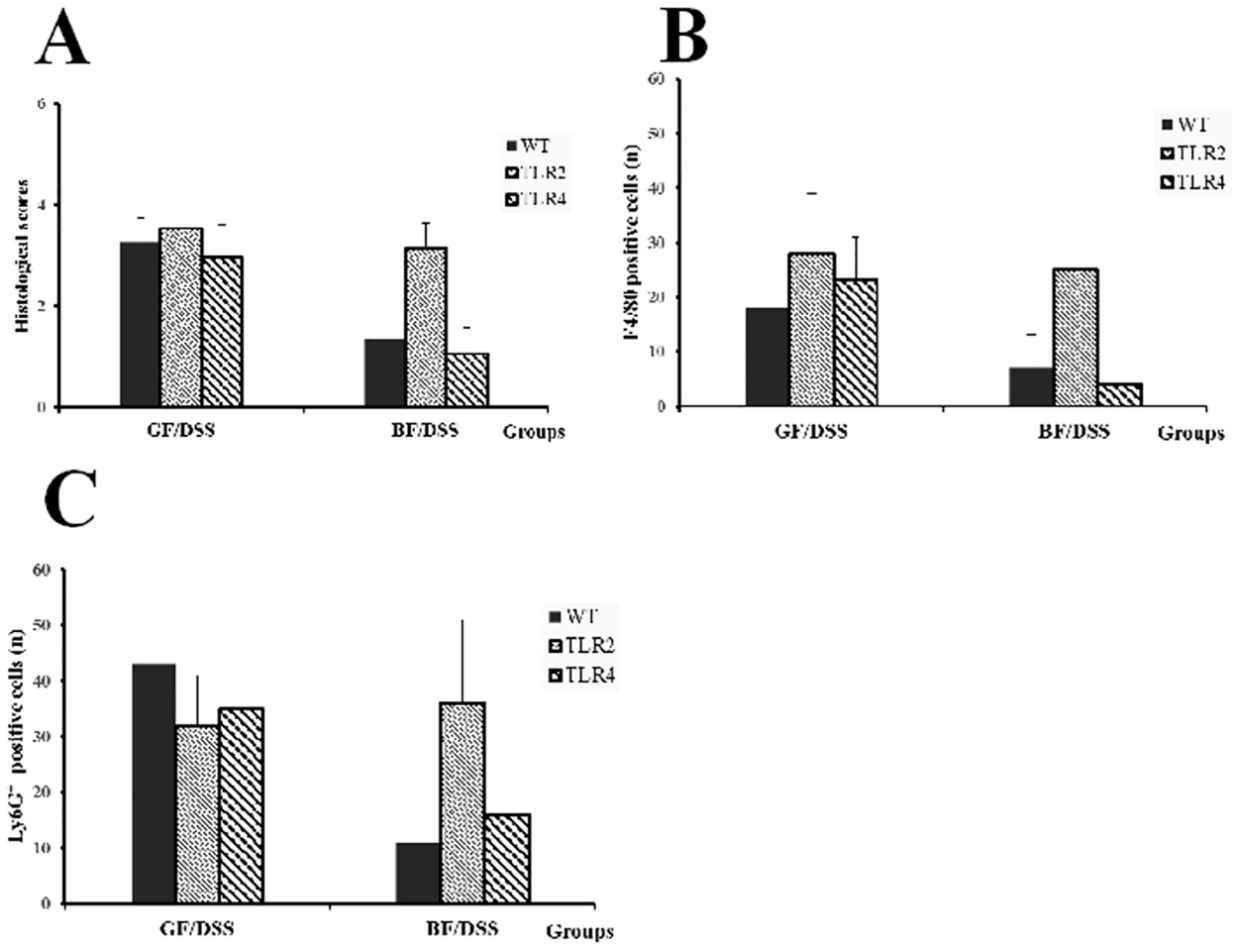
Histologic scores, F4/80 and Ly6G^+^ immunohistochemistry staining in DSS-treated WT, TLR4, and TLR2-mice under GF- or *BF*-condition. (A) Histological scores, (B) F4/80-positive cells, (C) Ly6G^+^-positive cells. *P < 0.05 compared to TLR2/*BF*/DSS.

In terms of colonic architecture, no difference was found between mice in the GF and *BF* groups (without DSS administration) (S1 Fig). In all GF groups, DSS treatment resulted in severe epithelial mucosa ulceration, basal crypt loss, goblet cell depletion, and significant leucocyte infiltration in the lamina propria and submucosa. In contrast, the colon tissue showed low/moderate inflammatory cell infiltration in the lamina propria with mostly intact mucosal architecture and slight submucosa edema in the WT/*BF*/DSS and TLR4/*BF*/DSS groups. In the TLR2/*BF*/DSS group, the crypt architecture was mostly disrupted, with extensive superficial ulceration and severe leucocyte infiltration in the lamina propria (Fig 3). Histological scores for tissue damage were significantly lower in the WT/*BF*/DSS and TLR4/*BF*/DSS groups than in the TLR2/*BF*/DSS group (1.33 ± 0.68 and 1.05 ± 0.52 versus 3.15 ± 0.49, respectively, *P* < 0.05) (Fig 4).

### Immunohistochemistry

**Fig 5:**
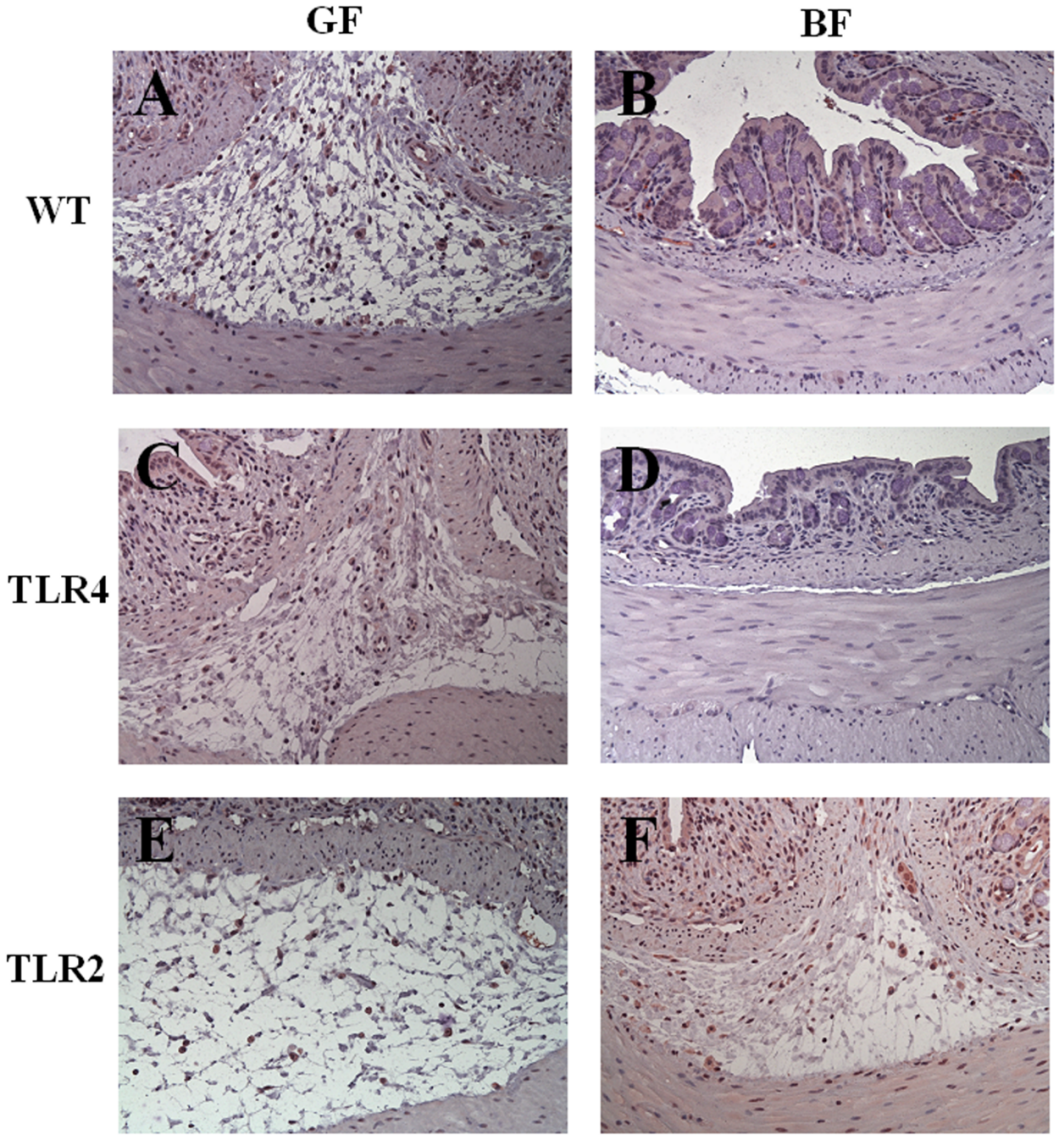
Immunohistochemistry staining for F4/80 (macrophages) in colon tissue sections. (A) WT/GF/DSS, (B) WT/*BF*/DSS, (C) TLR4/GF/DSS, (D) TLR4/*BF*/DSS, (E) TLR2/GF/DSS, and (F) TLR2/*BF*/DSS. Magnification ×200, bar = 20 *μ*m.

**Fig 6:**
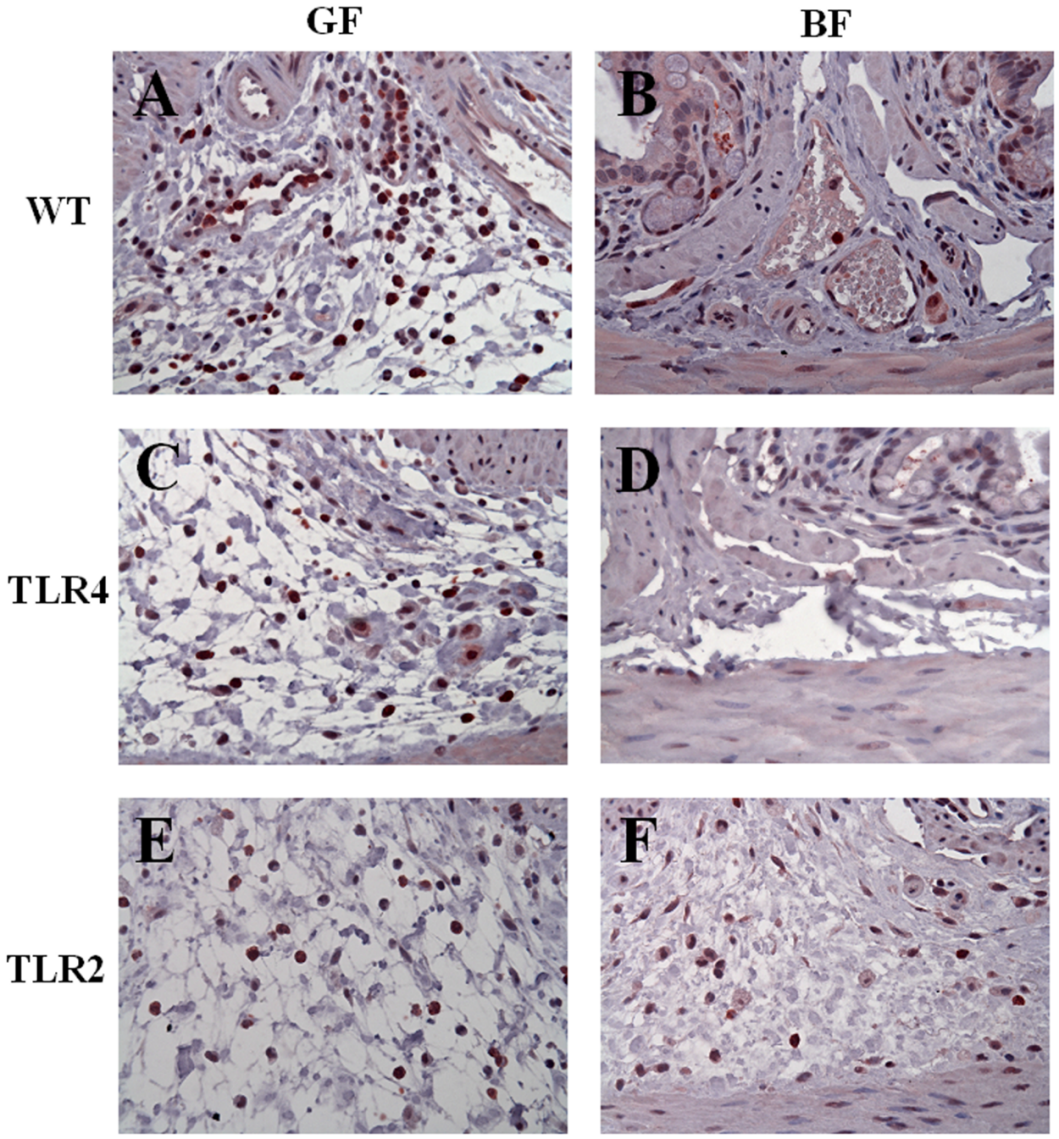
Immunohistochemistry (Ly-6G, granulocytes) evaluation of DSS-induced colitis in different knockout mice with or without *BF* monocolonization. (A) WT/GF/DSS, (B) WT/*BF*/DSS, (C) TLR4/GF/DSS, (D) TLR4/*BF*/DSS, (E) TLR2/GF/DSS, and (F) TLR2/*BF*/DSS. Magnification ×400, bar = 40 *μ*m.

The cellular composition of the inflamed colon was analyzed by immunohistochemical staining. The number of macrophages (F4/80) and granulocytes (Ly-6G) in the lamina propria and submucosa was assessed. In the GF condition, DSS induced a large number of F4/80-positive macrophages and Ly6G-positive neutrophils in the lamina propria in the WT/GF/DSS group. These results were consistent with those of our previous study. Under the GF condition, the number of F4/80- and Ly6G^+^-positive cells in the TLR4/GF/DSS and TLR2/GF/DSS groups was similar to in the WT/GF/DSS group (Figs 5 and 6). Interestingly, under the *BF* condition, the number of F4/80-positive (7 ± 6 and 4 ± 7 versus 25± 11) and Ly6G^+^-positive (11 ± 4 and 16 ± 9 versus 36 ± 15) cells was markedly decreased in the colon tissue of the WT/*BF*/DSS and TLR4/*BF*/DSS groups compared to in the TLR2/*BF*/DSS group, respectively (Fig 4).

### TNF-α, IL-6, and IL-10 mRNA expression in colon tissue

**Fig 7:**
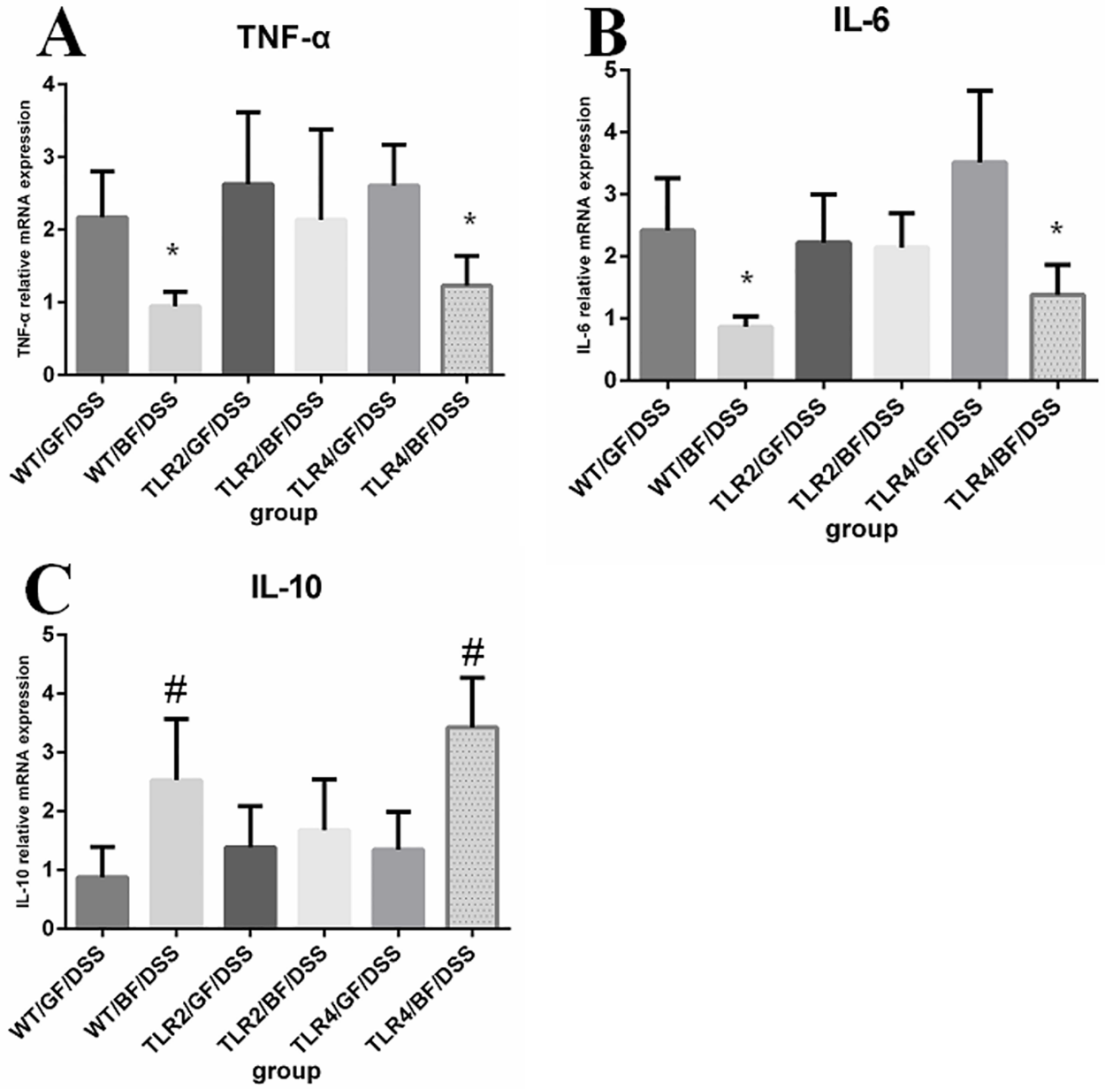
Expression profile of inflammatory and anti-inflammatory-related genes in colonic tissue of each group. (A) TNF-*α*, (B) IL-6 and (C) IL-10. *P < 0.05 compared to TLR2/*BF*/DSS; ^*#*^*p* < 0.05 compared to WT/GF/DSS.

As shown in S1 Fig, colonization of *BF* did not markedly affect TNF-α, IL-6, and IL-10 mRNA expression in colonic tissue. After administration of DSS, TNF-α mRNA levels were significantly increased in the WT/GF/DSS, TLR4/GF/DSS, and TLR2/GF/DSS groups compared to in the water only groups. The expression levels of TNF-α were significantly decreased in the WT/*BF*/DSS and TLR4/*BF*/DSS groups compared to in the TLR2/*BF*/DSS group. There was no difference in TLR2/GF/DSS compared to in the TLR2/*BF*/DSS group. The mRNA levels of IL-6 were significantly increased in all GF/DSS groups compared to the groups without DSS. However, the increase in IL-6 was attenuated in the WT/*BF*/DSS and TLR4/*BF*/DSS groups, but not in the TLR2/*BF*/DSS group. In contrast, the expression of IL-10 was increased in the WT/*BF*/DSS and TLR4/*BF*/DSS groups compared to in the WT/GF/DSS and TLR4/GF/DSS groups. There were no significant differences between the TLR2/GF/DSS and TLR2/*BF*/DSS groups (Fig 7).

### *BF* colonization could not ameliorate DSS-colitis in IL-10 knockout mice

**Fig 8:**
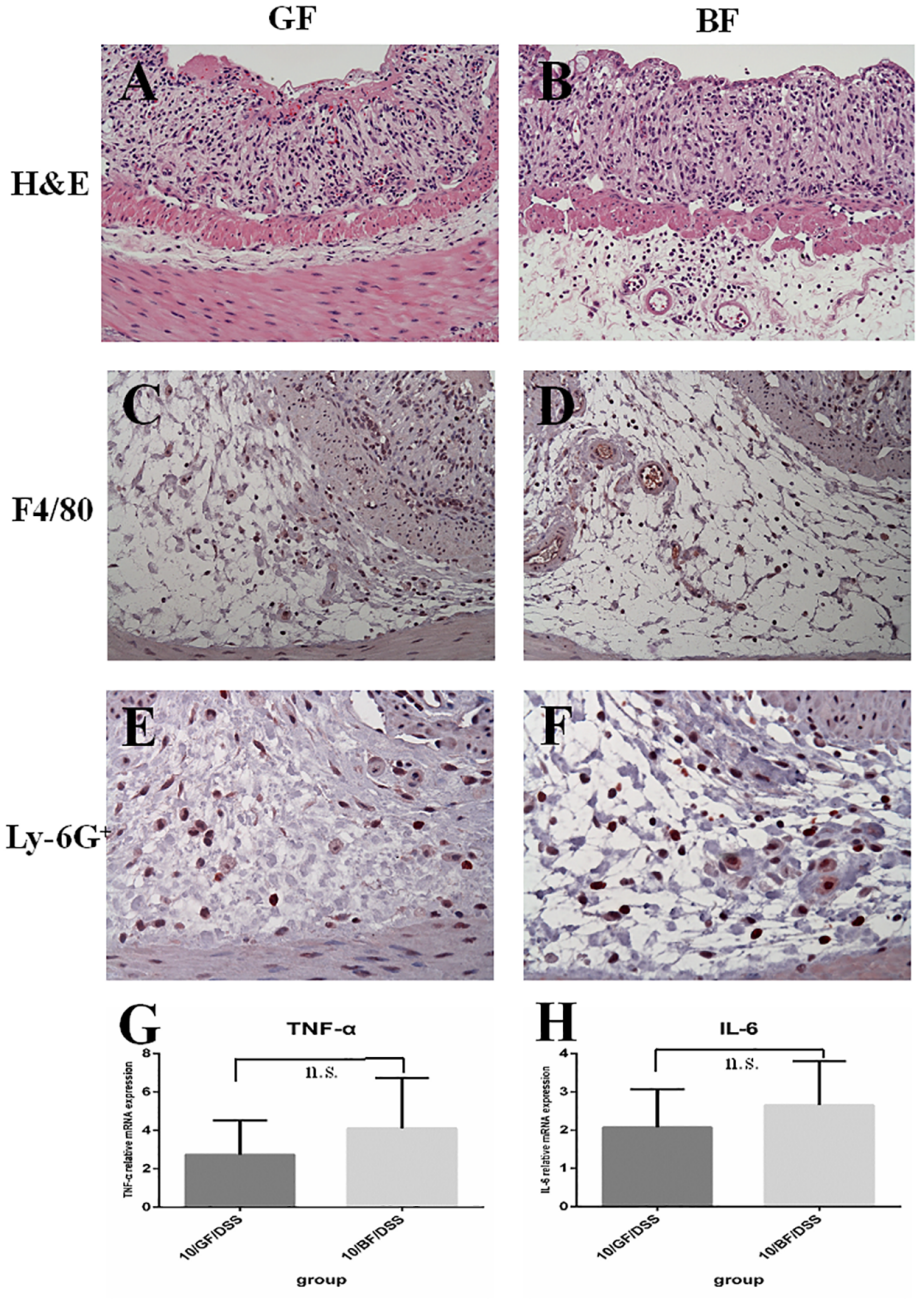
Histopathology and immunohistochemistry staining for IL10^-/-^ knockout mice with or without *BF*-treated DSS. (A) 10/GF/DSS, H&E (B) 10/*BF*/DSS, H&E (C) 10/GF/DSS, F4/80, (D) 10/*BF*/DSS, F4/80, (E) 10/GF/DSS, Ly-6G^+^, and (F) 10/*BF*/DSS, Ly-6G^+^, (G) levels of TNF-*α* in colonic tissue, (H) levels of IL-6 in colonic tissue. n.s.: statistically non-significant difference. Magnification ×400, bar = 40 *μ*m.

To further investigate the mechanism on *BF*-ameliorated DSS-colitis, IL-10 knockout mice were colonized with *BF* and then treated with 1% DSS in drinking water. Gross findings revealed only slight weight loss after inoculation with *BF* in 10/*BF*/WA mice. After 7 days of 1% DSS treatment, *BF* did not ameliorate DSS-induced fecal occult blood, colon shortening, elevated white blood cells, and colon ulceration in the 10/*BF*/DSS group compared to in the 10/GF/DSS group (Table 2). Positive inflammatory cells (F4/80 and Ly-6G^+^) were not improved in the 10/GF/DSS and 10/*BF*/DSS groups (Fig 8). In addition, the levels of inflammatory cytokines (TNF-α and IL-6) of colonic tissues in the 10/*BF*/DSS group were markedly higher than in the WT/*BF*/DSS group (*P < 0*.*05*).

**Table 2:** No significant differences in clinical observation, complete blood count, histology and immunohistochemical staining between 10/GF/DSS and 10/*BF*/DSS groups.

	10/GF/DSS	10/*BF*/DSS
Body weight (g)	32.5 ± 1.4	27.4 ± 1.2
Spleen weight (g)	0.064 ± 0.005	0.066 ± 0.007
Spleen/body weight (%)	0.20 ± 0.01	0.24 ± 0.03
Colon length (cm)	7.5 ± 1.0	7.7 ± 0.6
Occult index	3.5 ± 0.5	3.1 ± 0.2
Leukocytes (10^3^ cells/μL)	8.0 ± 1.2	7.9 ± 1.5
Neutrophils	0.8 0 ± 0.19	0.96 ± 0.33
Lymphocytes	3.30 ± 1.44	3.15 ± 0.85
Monocytes	0.09 ± 0.10	0.06 ± 0.03
Erythrocytes (10^3^ cells/μL)	9.7 ± 0.7	9.9 ± 0.6
Hemoglobin level (g/dL)	13.0 ± 0.8	13.7 ± 0.9
Platelet count (/μL)	47.0±3.6	48.7 ± 2.6
Histological scores	3.31 ± 0.52	3.17 ± 0.39
F4/80-positive cells (n)	24 ± 6	27 ± 8
Ly6G^+^-positive cells (n)	34 ± 5	39 ± 8

## Discussion

Innate immunity, particularly TLRs, plays an important role in intestinal homeostasis. A previous study reported a significant increase in TLR2 expression in the terminal ileum, whereas TLR4 expression was observed in the rectum of patients with UC [26]. However, detailed knowledge of the individual gut microbiota (such as the prevalence of *BF*), TLRs, and their relationship with UC remains limited. Our previous study demonstrated that monocolonization of *BF* ameliorated inflammation and immune cell influx into damaged gut tissue in a DSS-induced UC model [8]. Here, we determined that *BF* bacteria alleviated DSS-induced colitis through the TLR2 receptor but not TLR4. These results suggest that TLR2 is a crucial factor in intestinal physiological homeostasis. Our results provide important insights into a previously unrecognized role of *BF* in preventing DSS-induced colitis in GF mice.

To determine whether *BF* actively affects the homeostatic control of intestinal inflammation, we utilized TLR2 knockout mice colonized with *BF* and subsequently treated with DSS. Here, disease symptoms, complete blood count, and histopathology were not improved compared with WT mice of the same status. In contrast, the severity of colitis (including longer colon length and histologic damage score) was improved in TLR4 knockout mice colonized with *BF*. These results are similar to those of Lowe et al. who reported a protective role for TLR2 in epithelial injury, including the maintenance of epithelial homeostasis regulatory mechanisms in colitis-associated inflammation [20]. In addition, Burn et al. showed that TLR2 is significantly upregulated during IBD in patients and mice with DSS-induced colitis, indicating that changes in the expression of TLR2 and subsequent alterations in the innate immune response contribute to the pathogenesis of IBD [27]. These studies support the critical role TLR2 in protection from colitis initiation and progression. Moreover, TLR2 is activated by several microbial products, including peptidoglycans and lipoproteins from both Gram-positive and Gram-negative bacteria [28]. It is also the receptor for *BF* polysaccharide A, as described previously [29]. According to the above results and references, *BF* can alleviate DSS-induced colitis via the TLR2 signaling pathway.

In UC, the innate immune response induces inflammatory cells to secrete cytokines that play a critical role in disease progression [30]. Activated macrophages and neutrophils secrete proinflammatory cytokines such as TNF-*α*, IL-1*β*, and IL-6 to regulate the inflammatory response in the colonic mucosa of patients with UC [31–33]. In previous a study, we reported that colonization by *BF* decreased the production of the proinflammatory cytokine TNF-*α* and increased the anti-inflammatory cytokine IL-10 in the colon. It was also previously determined that *BF* influenced both the induction and effector functions of the mucosal immune system [8].

In the present study, we examined inflammatory and anti-inflammatory cytokine gene expression levels in TLR4/GF/DSS, TLR2/GF/DSS, and WT/GF/DSS groups under germ-free conditions. Gene expression of the pro-inflammatory cytokines TNF-*α* and IL-6 and the anti-inflammatory cytokine IL-10 were not significantly different in each group. These results are consistent with our hypothesis that different TLR genotypes do not affect the severity of DSS-colitis in the GF state. Interestingly, differences were observed in gene knockout mice (TLR4 and TLR2) colonized with *BF* and then administered with DSS. We observed significantly lower TNF-*α* mRNA expression, as well as a notable trend toward decreased IL-6 expression, in the TLR4/*BF*/DSS and WT/*BF*/DSS groups compared to the TLR2/*BF*/DSS group. Lorea Baroja et al. reported that *Lactobacillus rhamnosus* GR-1 and *L*. *reuteri* RC-14 significantly reduced TNF-α in the peripheral blood of IBD patients [34]. In addition, *Lactobacillus casei* has been shown to decrease TNF-α in the inflamed ileum of CD patients [35]. Our findings were similar to those of reports suggesting that probiotics down-regulate TNF-*α* and IL-6 production [36, 37]. *Bacteroides thetaiotaomicron* attenuation of proinflammatory cytokine expression by the active nuclear factor-κB pathway was also reported [38]. However, the pathway through which *BF* functions requires further investigation.

IL-10 is one of the most potent anti-inflammatory cytokines and is required for protection in many animal models of inflammation [13]. Based on our results, the transcriptional expression of IL-10 within the colon tissue of TLR4/*BF*/DSS and WT/*BF*/DSS groups was significantly higher than that in the TLR2/*BF*/DSS group. A recent publication also showed that *BF*-PSA enhanced the production of IL-10 in a trinitrobenzene sulfonic acid-colitis mouse model [13]. The gene expression of IL-10 was not significantly different between groups in the GF state. In addition, *BF* colonized IL-10 knockout mice, which were then challenged with 1% DSS for 7 days. *BF* could not prevent DSS-induced colitis in IL-10 knockout mice. Our results were similar to those of previous studies indicating that intestinal bacterial induces IL-10 activation and expression through TLR/nuclear factor-κB signaling mainly in mucosal immune cells to involve colitis [39]. These results suggest that *BF* inhibits the expression of proinflammatory cytokines including TNF-*α* and IL-6 and enhances anti-inflammatory cytokines such as IL-10 by regulating TLR2 signaling. Overall, our results suggest that *BF* improves DSS-induced colitis by inhibiting proinflammatory cytokines.

## Conclusion

These results indicate that the beneficial effects of *BF* in DSS-induced colitis are associated with alterations in the innate immune response through the TLR2/IL-10 signaling pathway.

## Supporting information

S1 Fig No difference was found between mice of the GF and *BF* groups without DSS treatment. (A) WT/GF, (B) WT/*BF*, (C) TLR4/GF, (D) TLR4/*BF*, (E) TLR2/GF, (F) TLR2/*BF* (G) 10/GF, and (H) 10/*BF*. H&E, magnification ×200, bar = 20 *μ*m.
(TIF)

S2 Fig Expression profile of inflammatory and anti-inflammatory related genes in the colonic tissue of each group. (A) TNF-*α*, (B) IL-6, and (C) IL-10.
(TIF)

S1 Table Gross finding in WT, TLR4, and TLR2 GF-mice with or without *BF* colonization. (DOCX)

S2 Table Effect of *BF* colonization on hematological characteristics in each group. (DOCX)
