# Supplementary figures and images for: TLR2 and interleukin-10 are involved in Bacteroides fragilis-mediated prevention of DSS-induced colitis in gnotobiotic mice
Source: PLoS One. 2017 Jul 6;12(7):e0180025. doi: 10.1371/journal.pone.0180025 (PMC5500315; doi:10.1371/journal.pone.0180025)

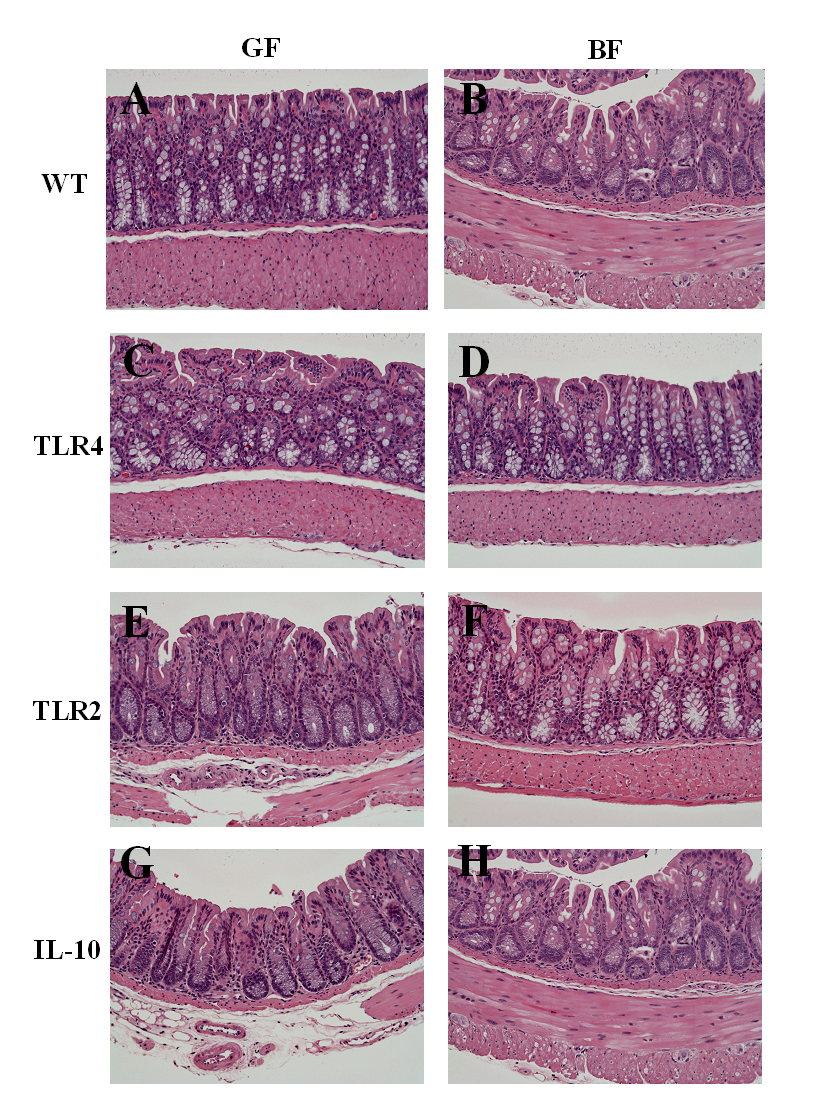

Supplement: S1 Fig — (A) WT/GF, (B) WT/BF, (C) TLR4/GF, (D) TLR4/BF, (E) TLR2/GF, (F) TLR2/BF (G) 10/GF, and (H) 10/BF. H&E, magnification ×200, bar = 20 μm. (TIF) [file pone.0180025.s001.tif]

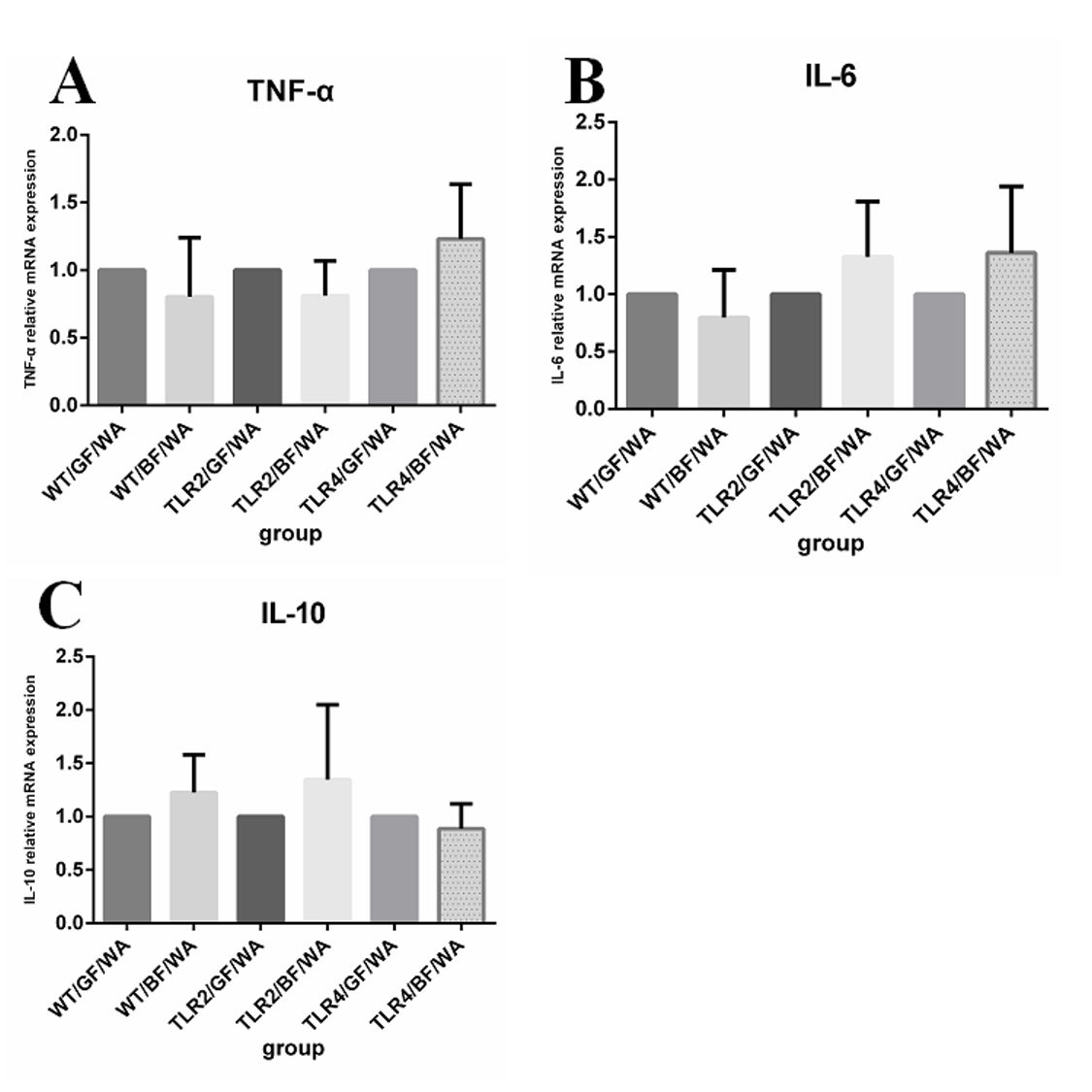

Supplement: S2 Fig — (A) TNF-α, (B) IL-6, and (C) IL-10. (TIF) [file pone.0180025.s002.tif]
