# Supplementary material for: TLR2 and interleukin-10 are involved in Bacteroides fragilis-mediated prevention of DSS-induced colitis in gnotobiotic mice
Source: PLoS One. 2017 Jul 6;12(7):e0180025. doi: 10.1371/journal.pone.0180025 (PMC5500315; doi:10.1371/journal.pone.0180025)
Supplement: S1 Table — (DOCX) [file pone.0180025.s003.docx]

**S1 Table.** **The gross finding in WT, TLR4 and TLR2 GF-mice with or without *BF* colonization.**

|  |  | GF/WA | | |  | *BF*/WA | | |
| --- | --- | --- | --- | --- | --- | --- | --- | --- |
|  |  | WT | TLR2 | TLR4 |  | WT | TLR2 | TLR4 |
| Body weight (g) |  | 27.1±2.1 | 29.0±0.8 | 30.4±1.7 |  | 27.8±2.0 | 26.9±1.2 | 29.4±1.6 |
| Spleen weight (g) |  | 0.064±0.007 | 0.065±0.015 | 0.064±0.006 |  | 0.063±0.012 | 0.058±0.005 | 0.057±0.007 |
| Spleen/body weight (%) |  | 0.24±0.03 | 0.23±0.02 | 0.21±0.01 |  | 0.23±0.04 | 0.22±0.03 | 0.19±0.03 |
| Colon length (cm) |  | 9.3±0.8 | 9.4±0.5 | 9.6±0.6 |  | 9.2±0.6 | 8.8±0.8 | 9.3±0.8 |
| Occult index |  | 0±0 | 0±0 | 0±0 |  | 0±0 | 0±0 | 0±0 |
