# Supplementary material for: TLR2 and interleukin-10 are involved in Bacteroides fragilis-mediated prevention of DSS-induced colitis in gnotobiotic mice
Source: PLoS One. 2017 Jul 6;12(7):e0180025. doi: 10.1371/journal.pone.0180025 (PMC5500315; doi:10.1371/journal.pone.0180025)
Supplement: S2 Table — (DOCX) [file pone.0180025.s004.docx]

**S2 Table. Effect of *BF* colonization on haematological characteristics in each group.**

|  |  | GF/WA | | |  | *BF*/WA | | |
| --- | --- | --- | --- | --- | --- | --- | --- | --- |
|  |  | WT | TLR2 | TLR4 |  | WT | TLR2 | TLR4 |
| Leukocytes (10^3^ cells/ul) |  | 7.2±1.4 | 7.1±1.9 | 6.2±1.2 |  | 6.4±0.8 | 7.2±1.4 | 5.7±1.1 |
| Neutrophils |  | 0.42±0.14 | 0.37±0.16 | 0.41±0.12 |  | 0.58±0.10 | 0.58±0.25 | 0.41±0.25 |
| Lymphocytes |  | 3.62±0.92 | 5.07±1.50 | 3.94±1.14 |  | 2.50±0.60 | 2.83±0.97 | 2.71±0.41 |
| Monocytes |  | 0.13±0.09 | 0.11±0.04 | 0.10±0.08 |  | 0.10±0.08 | 0.13±0.03 | 0.09±0.03 |
| Erythrocytes (10^3^ cells/ul) |  | 10.0±0.7 | 10.4±0.2 | 10.8±0.3 |  | 9.9±0.6 | 10.5±0.6 | 10.8±0.6 |
| Haemoglobin level (g/dl) |  | 14.6±1.0 | 15.3±0.4 | 15.4±0.5 |  | 14.6±0.7 | 14.8±0.9 | 15.2±0.7 |
| Haematocrit value (%) |  | 50.6±3.9 | 53.6±1.3 | 54.2±1.7 |  | 51.6±3.1 | 52.3±3.0 | 54.0±2.9 |
| Platelet count (/ul) |  | 1387.0±298.6 | 1212.3±178.5 | 1470.0±211.5 |  | 1160.6±147.0 | 1279.0±214.6 | 1362.1±283.2 |
